# Deciduous Dental Pulp Stem Cells for Maxillary Alveolar Reconstruction in Cleft Lip and Palate Patients

**DOI:** 10.1155/2020/6234167

**Published:** 2020-03-12

**Authors:** Daniela Y. S. Tanikawa, Carla C. G. Pinheiro, Maria Cristina A. Almeida, Claudia R. G. C. M. Oliveira, Renata de Almeida Coudry, Diógenes Laercio Rocha, Daniela Franco Bueno

**Affiliations:** ^1^Instituto Sírio-Libanês de Ensino e Pesquisa, Hospital Sírio-Libanês, São Paulo, SP, Brazil; ^2^Departamento de Fissura Lábio Palatina, Hospital Municipal Infantil Menino Jesus, São Paulo, SP, Brazil

## Abstract

**Background:**

To reduce morbidity to cleft patients, new approaches have been developed and here, we report for the first time the use of deciduous dental pulp stem cells (DDPSC) associated with a hydroxyapatite-collagen sponge (Bio-Oss Collagen® 250 mg, Geistlich) for closing alveolar defects during secondary dental eruption, further comparing these results to historical controls.

**Methods:**

Six patients, aged 8 to 12, were selected. Autologous DDPSC were isolated from each patient, then associated with the biomaterial and this bone tissue engineered set was used to fill the alveolar defect. Computed tomography was performed to assess both preoperative and 6- and 12-month postoperative outcomes. Overall morbidity was recorded. Historical controls consisted of sixteen patients previously selected and randomly assigned to group one (rhBMP-2) or group two (iliac crest bone graft).

**Results:**

DDPSC could be isolated and characterized as mesenchymal stem cells. Progressive alveolar bone union has occurred in all patients. Similarly to group two 75.4%, SD ± 4.0, *p* > 0.999, but statistically different from group one (59.6%, SD ± 9.9, *p* > 0.999, but statistically different from group one (59.6%, SD ± 9.9,

**Conclusion:**

For this selected group of patients, DDPSC therapy resulted in satisfactory bone healing with excellent feasibility and safety, which adds significantly to the prospect of stem cell use in clinical settings. *Clinical Question/Level of Evidence*. Therapeutic, II. This trial is registered with https://clinicaltrials.gov/ct2/show/NCT01932164?term=NCT01932164&rank=1.

## 1. Introduction

To overcome donor site morbidity during secondary maxillary alveolar reconstruction in cleft lip and palate (CLP) patients, many innovative efforts regarding various bone substitutes have been reported [1, 2].

However, the lack of bioactivity, biomechanical weaknes, and susceptibility to infection are still detrimental to the use of most of them; [3] and even for bone morphogenetic proteins, recently suggested as an effective alternative [4–7], significant restraints concerning high costs and severe adverse events have emerged [8–11].

**Figure 1:**
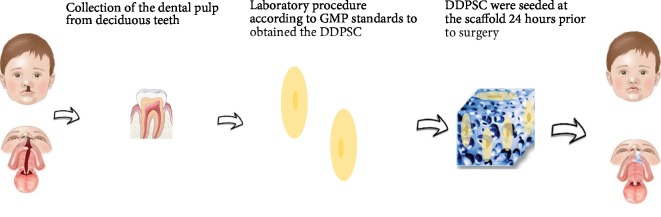
Representative strategy for autologous bone tissue engineering using DDPSC.

Diversely, tissue engineering strategies arise as a new therapeutic option and one of the research hotspots in recent years [2, 12–16]. Therefore, considering that during the mixed dentition every child has deciduous exfoliating teeth, we decided to carry out a phase 1 clinical study using deciduous dental pulp stem cells (DDPSC) for maxillary alveolar cleft reconstruction (Figure 1) [17, 18].

The results of this prospective cohort of patients were then compared to historical controls, which received either the traditional iliac crest bone graft or bone morphogenetic protein 2 (rhBMP-2) [6]. Outcomes of interest were the alveolar cleft bone filling, the new bone's ability to withstand dental eruption, and the occurrence of complications.

## 2. Methods

### 2.1. Study Design

This study is a retrospective review of a prospective cohort and a historical group of patients who previously had been submitted to a randomized, controlled, observer-blinded, and surgical trial with two parallel comparison groups.

### 2.2. Trial Population and Eligibility Criteria

For this phase I trial, six patients with unilateral alveolar cleft defects, aged 8 to 12 years old, were selected at Hospital Municipal Infantil Menino Jesus (HMIMJ). All patients underwent preoperative orthodontic expansion of maxillary segments. Exclusion criteria used were previous alveolar surgery, previous eruption of the canine, presence of comorbidities, or incomplete records. Informed consent was obtained for each subject at study entry (written and verbal guidelines).

### 2.3. Interventions

Patients underwent maxillary alveolar reconstruction using a hydroxyapatite-collagen sponge (Bio-Oss Collagen® 250 mg, Geistlich) associated with DDPSC (*N* = 6). Historical controls met the same eligibility criteria and consisted of subjects who previously underwent maxillary alveolar reconstruction with either rhBMP-2 (group one, *N* = 8) or the traditional iliac crest bone graft (group two, *N* = 8).

### 2.4. Collection and Expansion of DDPSC

Extraction of the deciduous teeth was performed at the dental office at HMIMJ. By endodontic removal, the deciduous tooth pulps were obtained and immediately added to a sterile collector with 2 mL DMEM-F12 solution (Dulbecco's Modified Eagle Medium/Nutrient Mixture F12, Gibco Invitrogen, Grand Island, NY). Then, they were transported to the laboratory of good practices of manipulation (GMP) at Centro de Tecnologia Celular-Hospital Sírio-Libanês (HSL) up to 24 hours after the collection. With the use of a solution containing 1 mg/mL trypsin (TrypLE, Gibco Invitrogen, Grand Island, NY) in PBS (pH 7.4, Gibco Invitrogen, Grand Island, NY) for 30 minutes at 37°C, stem cells were obtained. Then, pulp tissues were fragmented and cultured in Dulbecco Modified Water/Nutrient Modules F12 (Gibco, Grand Island, NY) supplemented with 15% bovine fetal serum (HyClone, GE Health Care Life Sciences), preferably distributed in different wells and maintained with DMEM-F12 medium (Gibco, Grand Island, NY), 2% NEAA (MEM nonessential amino acid solution, Gibco, Grand Island, NY), and 2% penicillin and streptomycin (Gibco, Grand Island, NY) and incubated at 37°C in an atmosphere of 10% CO_2_. Culture medium was changed every three days, and only the DPSC that had been cultured for three to five passages were used in this study.

### 2.5. Identification and Characterization of DDPSC

The cellular characterization was performed by flow cytometry on FACSCalibur (BD Biosciences, Becton Dickinson, Franklin Lakes, NJ) and analyzed in their own CellQuest program (BD Biosciences). Cells obtained from cell cultures at a concentration of 1 × 10^6^ cells in 100 *μ*L were labeled with monoclonal antibodies—CD29-PE, CD31-FITC, CD34-FITC, CD44-PE, CD45-PE, CD73-FITC, CD90-FITC, CD105-PE, IgG-FITC, and IgG-PE (BD Biosciences)—for 30 minutes at room temperature, in the dark.

Next, DDPSC were tested for their ability to differentiate into adipocytes, chondrocytes, and osteoblasts in accordance to the methods previously described [17, 18]. To analyze the presence of aerobic, anaerobic bacteria, and fungi in the culture, the automated microbial detection system BacT/Alert 3D (BacT/Alert-bioMérieux-Durham, NC) was used. Any positive samples were discharged, and then, new dental pulp collection was recommended.

### 2.6. Tissue-Engineered Bone Graft

One million DDPSC were seeded at 250 mg Bio-Oss Collagen® (Geistlich Biomaterials AG, Wolhusen, Germany) 24 hours prior to surgical procedure, and for each patient, two to four sets were prepared.

### 2.7. Electron Microscopy

Cells were fixed at the biomaterial 250 mg Bio-Oss Collagen® (Geistlich Biomaterials AG, Wolhusen, Germany) during 24 hours at 4°C using the modified Karnovsky solution, which contains 2.5% glutaraldehyde and 2% paraformaldehyde in 0.1 M (pH 7.4) sodium cacodylate buffer. The specimens were postfixed in 2% osmium tetroxide solution and rinsed in distilled water for one hour at room temperature. Then, the cells were dehydrated using ethanol and cleansed in an ultrasonic apparatus (Gerador DA 200, Thornton Inpec Eletronica SA, São Paulo, SP, Brazil) for one hour. The samples were mounted on metal stubs and covered with gold in a sputter coater apparatus (Balzers Union SCD-040, Liechtenstein). The specimens were examined in a scanning electron microscope at 20.0 kV (Jeol 6460LV, Tokyo, Japan).

### 2.8. Surgical Procedure

The surgical procedure and the alveolar defect exposure were performed in the same standardized manner and by the same surgical team of historical controls, as previously described [6]. In accordance with its size, two to three bone tissue-engineered sets, each one composed of 250 mg Bio-Oss Collagen® (Geistlich Biomaterials AG, Wolhusen, Germany) with one million DDPSC, were placed into the alveolar defect.

For the historical controls, the 2.8 mL kit of Infuse® Bone Graft (Medtronic, Memphis, TN) with a dose range of 3.2 to 4.2 mg was used as bone morphogenetic protein source in group one and in group two, a volume range of 20 to 40 mL of iliac crest cancellous bone was removed and applied into the defect.

### 2.9. Clinical Assessment

Preoperative variables included age, sex, and CLP classification. Length of hospital stay was recorded. Postoperatively, patients were asked to return for follow-up appointments on the 1^st^, 2^nd^, and 3^rd^ weeks and on the 6^th^ and 12^th^ months. On each follow-up visit, surgical complications such as bleeding, infection, oronasal fistula, bone graft exposure, or signs of ectopic bone formation were assessed and recorded.

### 2.10. Radiographic Assessment

For bone healing assessment, a computed tomography (CT) was performed, using Somatom Force AF2 Siemes Healthcare GmbH, Munich, Germany, preoperatively as well as on the 6^th^ and 12^th^ months postoperatively, as previously described [6]. A volumetric analysis of the alveolar defect on both cleft and noncleft sides was performed through the Osirix Dicom Viewer (Apple Inc. Website). By superimposing the coordinates on anatomical landmarks, preoperative and 6-and 12-month postoperative images were adjusted, unified, and compared. Anatomical landmarks were the pyriform aperture superiorly and the cementoenamel junction inferiorly. The difference between preoperative and postoperative defect volume was defined as the bone filling volume, and the percentage ratio between the bone filling volume and the preoperative defect volume was defined as the bone filling percentage. Dental eruption was assessed 12 months postoperatively.

### 2.11. Statistical Analysis

Statistical and inferential analyses were performed through the Statistical Package for the Social Sciences software (SPSS for Windows 13). The assumptions of normal distribution in each group and the homogeneity of variances between groups were evaluated, respectively, with Shapiro-Wilk and Levene's tests. In all inferential analysis a type I (*α*) error probability of 0.05 was considered. A statistical analysis of the data was carried out using the ANOVA for linear mixed effects models followed by the Bonferroni method.

## 3. Results

### 3.1. Characterization of DDPSC

Mesenchymal stem cells were isolated from dental pulps, showing fibroblast-like morphology. Flow cytometry analysis showed positive reactions for mesenchymal markers (CD29, CD44, CD73, CD90, and CD105) and negative reactions for endothelial (CD31) and hematopoietic markers (CD34 and CD45) (Table 1).

**Figure 2:**
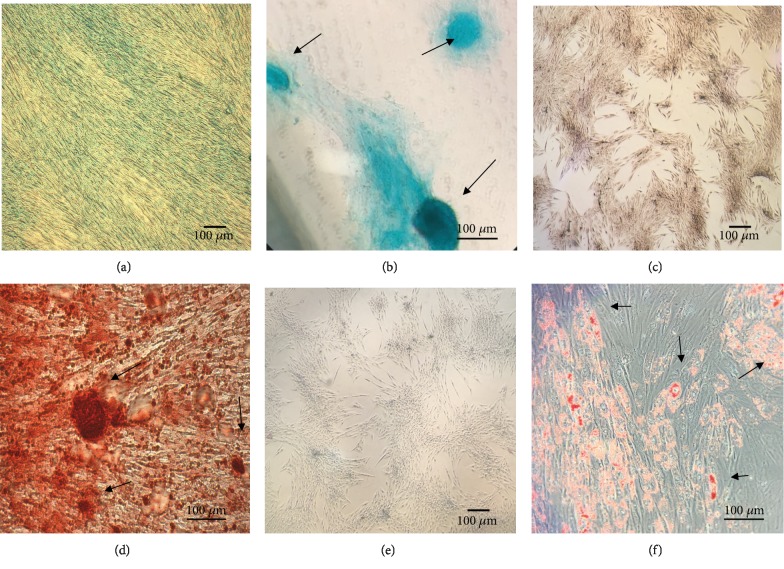
Multilineage differentiation “in vitro”. (a) The control group of DDPSC chondrogenic differentiation. (b) Chondrogenic differentiation after three weeks of DDPSC induction stained with Alcian Blue; black arrows show the extracellular matrix formation—mucopolysaccharides. (c) The control group of DDPSC osteogenic differentiation. (d) Osteogenic differentiation after three weeks of DPSC induction stained with Alizarin Red S; the black arrows show the calcium nodules. (e) Control group of DDPSC. (f) Adipocytes stained with Oil Red obtained after the adipogenic induction of DPSC during 18 days; black arrows show the fat vesicles. All the scale bars represent 100 *μ*m.

DDPSC were also characterized by inducing cellular differentiation into bone, cartilage, and fat. This was observed in all strains, demonstrating their multipotent capability (Figure 2).

### 3.2. Electron Microscopy

**Figure 3:**
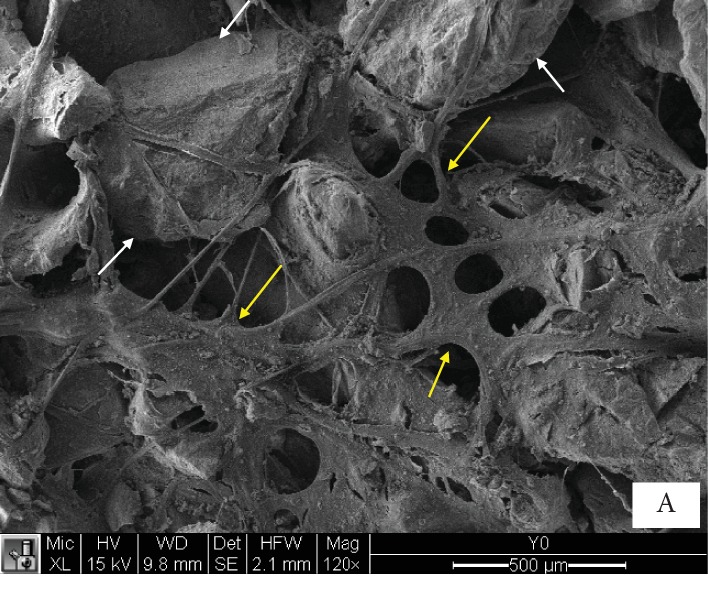
Electron microscopy. Scaffold 250 mg Bio-Oss Collagen® (Geistlich Biomaterials AG, Wolhusen, Germany) seeded with DDPSC; yellow arrows show DDPSC, and white arrows show the scaffold (Bio-Oss®).

Good attachment of DDPSC to the scaffold 250 mg Bio-Oss Collagen® (Geistlich Biomaterials AG, Wolhusen, Germany) could be routinely detected by electron microscopy (Figure 3).

### 3.3. Study Population and Surgical Variables

Six patients were enrolled in the study (three males and three females). Three patients had complete unilateral clefts, and the others had unilateral cleft lip and alveolus. Mean age at surgery was ten years and two months old (range 8 to 12). All these variables were comparable to historical controls.

### 3.4. Morbidity

For patients receiving DDPSC, there were no surgical complications. In group one, 37.5% developed significant swelling in the early postoperative period, and in group two, 87.5% complained about significant donor site pain at week two. Mean length of stay was longer for group two at day three, compared to patients receiving dental pulp stem cells and group one at day one postoperatively.

### 3.5. Bone Healing

**Figure 4:**
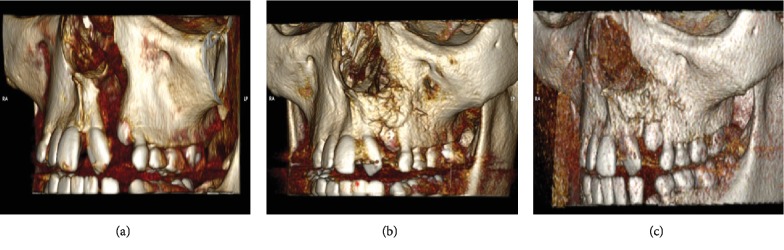
Patient that has received alveolar bone tissue engineering graft (DDPSC associated with 250 mg Bio-Oss Collagen®). Computed tomography images of the same patient showing the alveolar cleft fill by bone 6 and 12 months after the use of bone tissue engineering strategies (DDPSC associated with Bio-Oss Collagen®) and the canine tooth eruption after 12 months. (a) Preoperative—presence of alveolar cleft; (b) 6 months postoperatively—the alveolar cleft filled by new bone, and (c) 12 months postoperatively—canine tooth eruption in the new bone formed using the DDPSC associated with Bio-Oss Collagen®.

Preoperative and follow-up examinations revealed progressive alveolar bone union in all 6 patients that has received bone tissue engineering alveolar bone graft (DDPSC associated with 250 mg Bio-Oss Collagen®. Partial or total graft loss, wound breakdown, or ectopic bone formation were not observed in all patients that has received DDPSC associated with 250 mg Bio-Oss Collagen® (Geistlich Biomaterials AG, Wolhusen, Germany), Figure 4.

**Figure 5:**
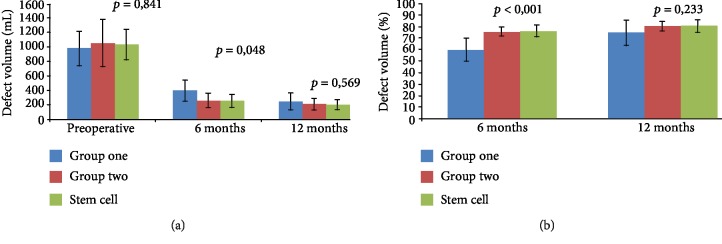
Analysis of volumetric and bone filling. (a) Volumetric representative graph. (b) Bone filling representative graph.

Through volumetric analysis, mean preoperative defect was 1028.6 mm^3^ (SD 212.6), resembling groups one and two (*p* = 0.841). At the 6-month follow-up examination, mean postoperative defect was 253.2 mm^3^ (SD 85.8) in group one and in group two 260.4 mm^3^ (SD 98.5); it was smaller than that in group one (393.6 mm^3^, SD 144.7, *p* = 0.048). However, at the 12-month follow-up examination, mean postoperative defect became similar in all groups (*p* = 0.569) (Figure 5(a) and Table 2).

Regarding bone filling percentage, there was a significant difference at the 6-month follow-up between patients receiving DDPSC (75.6%, SD 4.8) and group one (59.6%, SD 9.9, *p* = 0.001), but at the 12-month follow-up examination, this difference disappeared (*p* = 0.233) (Figure 5(b) and Table 3).

**Figure 6:**
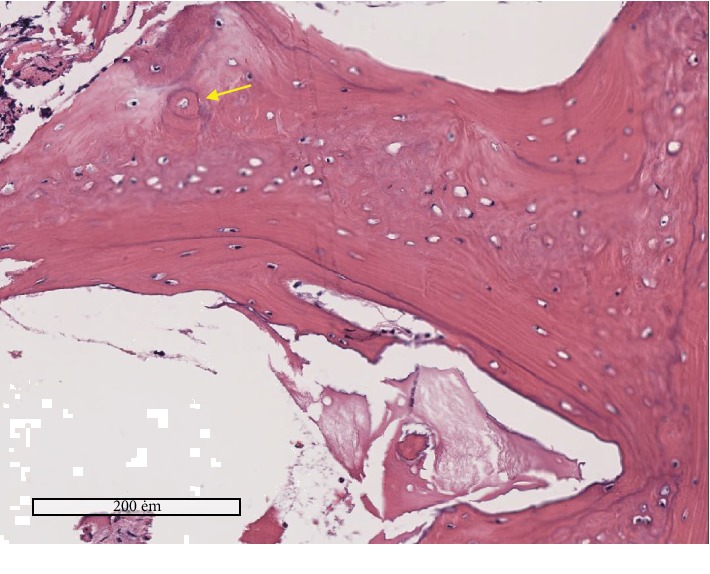
Histology of the bone formed with DDPSC associated with Bio-Oss Collagen® after 12 months. Histology of the bone formed after 12 months of the use of bone tissue engineering strategy (DDPSC associated with Bio-Oss Collagen® (Geitlish) to close the alveolar bone cleft of cleft lip and palate patients. The scale bars represent 200 *μ*m. The yellow arrow shows the presence of young bone (Haversian canal), and the black arrows show the presence of remaining biomaterial (Bio-Oss Collagen®, Geitlish) that was not reabsorbed in twelve months.

The patients biopsy twelve months after the use of bone tissue engineering to do their alveolar cleft rehabilitation using DDPSC associated with Bio-Oss Collagen® showed in the histology the presence of good young bone with only some reminiscent of the biomaterial (Bio-Oss®, Geitlish), Figure 6.

Dental eruption routinely occurred with 66.7% of patients. Canine impingement was detected in two patients, for which the canine tooth was drawn using orthodontic strategies. In groups one and two, no adverse event regarding dental eruption was detected.

## 4. Discussion

For the past two decades, the advent of bone tissue engineering represents a very promising alternative that circumvents several limitations of autografting. Currently, there are vigorous investigations on new strategies such as gene therapy, stem cells, and osteoinductive growth factors, but so far, only small series of patients in few controlled human clinical trials describe its use in the craniofacial surgical field [2, 5–7, 15, 16, 19–21].

For instance, the discovery of rhBMP-2 has prompted a spurt of activity to apply this growth factor into a variety of bone defects. Primarily observed in embryonic and skeletal development, small amounts of these proteins are found in mature skeletons for bone repair and maintenance [22].

However, for the recombinant human forms currently available, superphysiological doses with approximately 200,000 times the naturally occurring dose are detected. Thus far, rhBMP-2 has been approved by the US Food and Drug Administration (FDA) for autograft replacement for interbody spinal fusion, treatment of orthopedic trauma, sinus floor augmentations, and localized alveolar ridge augmentations for tooth extract defects in skeletally mature patients [23]. Several studies have shown successful rhBMP-2-induced bone formation in the craniofacial skeleton [5–7]. However, in spite of some exciting data from these human reports, major complications, adverse events, and reoperations have increasingly been attributed to the “off-label” use of rhBMP-2 in spine surgery, including heterotopic ossification, osteolysis, increased neurological deficits, and cancer, [9, 10, 24, 25] and for maxillofacial surgery, Neovius et al. and Goss et al. reported severe swelling, while high rates of postoperative nasal stenosis were described in cleft children [8, 11]. Because of this, a second FDA warning was issued against the use of this product in the pediatric population out of concern for insufficient data to demonstrate long-term efficacy or safety in children.

Diversely, tissue engineering strategies using scaffolds and mesenchymal stem cells are potential treatments for filling bone defects in the growing craniofacial skeleton, including alveolar clefts. For alveolar bone tissue engineering, the first clinical use was published in 2006 [12], and to date, few cases have been reported [2, 13–15]. After using osteoblasts cultured on demineralized bone matrix Osteovit® (Braun, Melsungen, Germany), Pradel and Lauer reported that 40.9% of the cleft defect was ossified [15]. In 2012, mesenchymal stem cells on biphasic scaffolds Reprobone® (Ceramisys, Sheffield, England) resulted in 51.3% of bone filling three months postoperation; [14] in Bajestan's study, cell therapy was successful in two out of five subjects [16]. In all these reports, less than half of the bone's defect was filled; in addition, the bone marrow had been the stem cell donor, which implies that their acquisition required prior harvesting procedure with drawbacks in both time and patient comfort.

In this study, we report for the first time the use of DDPSC for maxillary alveolar reconstruction in CLP patients. With similar properties and differentiation abilities to those derived from bone marrow [26], proangiogenic properties [27], and adipogenic, myogenic, neurogenic, and odontogenic potential [17, 26], DDPSC are easily accessible with very little morbidity from deciduous teeth during the mixed dentition. Besides, from only one deciduous tooth, it is possible to obtain 1 × 10^4^ DDPSC, and after five passages, that the number turns into 1 × 10^20^ cells. Considering that an average of two to three biomaterials embedded with 1 × 10^6^ cells was used in this study, we highlight that the number of cells needed could be easily obtained in approximately one month after dental extraction, which is a short period. Furthermore, in this study, we could observe by electronic microscopy that these DDPSC have a good interaction and adhesion to the biomaterial (Bio-Oss collagen®; Geistlich Biomaterials AG, Wolhusen, Germany).

Regarding scaffolding matrices, we previously observed that the collagen sponge carrier of rhBMP-2 lacked structural stability, causing the collapse of soft tissue walls in the grafted area [6]. Thus, to aid in maintaining bone induction space to occur during new bone formation, a hydroxyapatite-collagen sponge was selected for this study. Hydroxyapatite has inherent osteoconductive and osteoinductive properties; therefore, a hydroxyapatite-collagen sponge mixture takes advantage of the architectural strength of hydroxyapatite and the rapid dissolution profile of collagen. The Bio-Oss collagen® (Geistlich Biomaterials AG, Wolhusen, Germany) is characterized by a sponge structure and interconnected pore system that may facilitate cell adherence and vascular in-growth [28, 29]. However, due to its high degree of radiolucency, it cannot be detected by X-ray or CT examination until it has been replaced by autogenous bone. Our experimental study in minipigs, which employed CT, histologic, histomorphometric, and immunohistochemical analyses, provides evidence that the hydroxyapatite-collagen scaffolds seeded with DDPSC are more proficient than unseeded scaffolds for osteogenesis and new bone formation. These data unequivocally demonstrate that the addition of stem cells to a bone-mimetic biomaterial can improve the regenerative capacity of the tissue-engineered bone (unpublished data).

Objective parameters such as bone volume, labiolingual morphology, and bone architecture were assessed using the Osirix Dicom Viewer software (Apple Inc.). In all patients, it was detected that progressive alveolar bone union successfully occurred. However, while grafted cancellous bone is quickly incorporated and vascularized, being anatomically indistinguishable by CT at the 6-month follow-up examination, in the stem cell group, a slow resorption rate of the hydroxyapatite component was verified and at the 6 and 12-month follow-up examinations and small amounts of this product could still be detected. Still, the volume increase between 6 and 12 months postoperative suggests that this volume was replaced by autologous bone. Furthermore, when specifically evaluating interalveolar bone height and the distance between the apex and cementoenamel junction of both mesial and distal teeth, we detected a bone bridge formation with bone mineralization greater than 75% in all patients receiving stem cell therapy.

Nevertheless, for the stem cell group, canine impingement was observed in 33% of patients. In the literature, it has been shown that patients with alveolar clefts have a 20-fold increased risk for canine impaction based on canine position compared with the reported 1-2% prevalence of impacted canines in the general population [30, 31]. Therefore, these results are in accordance to recent reported rates of 35% and 18·9% for canine impaction in patients with clefts [32, 33].

Despite the lack of biomechanical analysis, we believe that a predisposing factor for canine impingement might be the slow resorption rate of the hydroxyapatite component, and not an unusual hardness of the regenerated bone, as suggested by Giuliani et al. [34] Three years after transplants in human mandibles, they showed that collagen sponge seeded with DPSC regenerated a compact rather than a spongy bone. In this study, 12 months postoperatively, bone biopsies could be collected from the treated sites in two patients: one from the stem cell group and the other from the iliac crest bone graft group. For both, the histological analysis demonstrated complete mineralization of the new bone and its integration with the intact bone boundaries, without any other difference between them, except for the small amounts of hydroxyapatite still present in the stem cell patient.

No ectopic bone growth related to the immature skeleton was detected in this study, whereas some investigative reports of ectopic bone formation have been associated with rhBMP-2. Hence, in this preliminary report, bone formation induced by DDPSC appears to have similar behavior to iliac crest bone engraftment, but canine impaction needs to be further investigated.

Many investigators are concerned about the potential complications of stem cell therapy. Therefore, we kept monitoring clinical parameters and, thus far (for up to five years), no complications or hazards associated with this new therapy modality have been detected.

As the first clinical report of alveolar bone tissue engineering using DDPSC in children, our study has the limitation of a small sample size in a series of patients. Despite the comparison to a historical cohort, randomized controlled trials are still necessary to substantiate the evidence for this strategy in various clinical circumstances.

## 5. Conclusion

In conclusion, we demonstrated that stem cell therapy results in satisfactory bone regeneration with dental eruption and reduced morbidity compared to traditional iliac crest bone grafting and rhBMP-2. These observations point out that stem cells can be potentially applied in reconstructing other insults in the craniofacial surgical field. Particularly, when it comes to rehabilitating the alveolar bone in CLP patients, the use of the DDPSC has the advantage of eliminating the need for a second surgical intervention (to obtain the iliac crest bone graft), thus having the potential to reduce operative time, intraoperative blood loss, postoperative pain, costs, and length of hospital stay—factors that could render regenerative medicine a reliable alternative for the current cleft care.

## Figures and Tables

**Table 1:** Immunophenotype profile (% positive reaction).

Patient ID	CD29	CD31	CD34	CD44	CD45	CD73	CD90	CD105
1	80	0.8	0.26	97.6	0.09	89.5	98.06	88
2	80	0.7	0.06	97.5	0.5	94.48	95.7	94.21
3	94	0.65	0.31	98.43	0.5	90.38	96.48	96.45
4	90.58	0.05	0.08	80	0.02	97.46	99.68	98.17
5	95.23	2	0.03	97.94	0.14	80	90	98.2
6	95.12	0.81	1.17	83.43	1.06	81.09	92.83	98.52

**Table 2:** Defect volume analysis.

	Preoperative		6-month follow-up		12-month follow-up	
	Mean (mm^3^)	SD	Mean (mm^3^)	SD	Mean (mm^3^)	SD
Group one (*N* = 8)	974.8	236.8	393.6	144.7	247.1	112.8
Group two (*N* = 8)	1052.4	326.0	260.4	98.8	207.8	77.9
Stem cell (*N* = 6)	1028.6	212.6	253.2	85.8	200.2	67.0
*p*	0.841		**0**.**048**^∗^		0.569	

SD: standard deviation. ^∗^Statistically significant difference.

**Table 3:** Bone filling percentage analysis.

	6-month follow-up		12-month follow-up	
	%	SD	%	SD
Group one (*N* = 8)	59.6	9.9	74.4	10.8
Group two (*N* = 8)	75.4	4.0	80.2	4.1
Stem cell (*N* = 6)	75.6	4.8	80.4	5.3
*p*	**<0**.**001**^∗^		0.233	

^∗^Statistically significant difference.

## Data Availability

The corresponding author had full access to all the data in the study and had final responsibility for the decision to submit for publication. Please contact author for data requests.

## Abbreviations

rhBMP-2: Recombinant human bone morphogenetic protein-2
CLP: Cleft lip and palate
DDPSC: Deciduous dental pulp stem cells
CT: Computed tomography
HMIMJ: Hospital Municipal Infantil Menino Jesus
SPSS: Statistical Package for the Social Sciences software
CD: Cluster of differentiation
DMEM-F12: Dulbecco's Modified Eagle Medium/Nutrient Mixture F12
SD: Standard deviation
PROADI-SUS: Programa de Apoio ao Desenvolvimento Institucional do SUS.
